# Combining effects from rare and common genetic variants in an exome-wide association study of sequence data

**DOI:** 10.1186/1753-6561-5-S9-S44

**Published:** 2011-11-29

**Authors:** Hugues Aschard, Weiliang Qiu, Bogdan Pasaniuc, Noah Zaitlen, Michael H Cho, Vincent Carey

**Affiliations:** 1 1Department of Epidemiology, Harvard School of Public Health, 677 Huntington Avenue, Boston, MA 02115, USA; 2 2Channing Laboratory, Brigham and Women’s Hospital, 181 Longwood Avenue, Boston, MA 02115, USA

## Abstract

Recent breakthroughs in next-generation sequencing technologies allow cost-effective methods for measuring a growing list of cellular properties, including DNA sequence and structural variation. Next-generation sequencing has the potential to revolutionize complex trait genetics by directly measuring common and rare genetic variants within a genome-wide context. Because for a given gene both rare and common causal variants can coexist and have independent effects on a trait, strategies that model the effects of both common and rare variants could enhance the power of identifying disease-associated genes. To date, little work has been done on integrating signals from common and rare variants into powerful statistics for finding disease genes in genome-wide association studies. In this analysis of the Genetic Analysis Workshop 17 data, we evaluate various strategies for association of rare, common, or a combination of both rare and common variants on quantitative phenotypes in unrelated individuals. We show that the analysis of common variants only using classical approaches can achieve higher power to detect causal genes than recently proposed rare variant methods and that strategies that combine association signals derived independently in rare and common variants can slightly increase the power compared to strategies that focus on the effect of either the rare variants or the common variants.

## Background

Genome-wide association analysis of common DNA variants (usually single-nucleotide polymorphisms [SNPs]) has been successful in finding common variants associated with complex diseases and phenotypes. However, most of these associated variants have small effect size, and thus the proportion of heritability explained is usually modest. An increasingly popular suggestion to address this issue is to shift attention from searching for common variants of small effect to searching for rare variants with larger effects [1]. However, complex diseases can be influenced by both common and rare variants within the same gene [2]. Although many methods for analyzing common variants have been proposed and have proved successful in identifying loci associated with phenotype, recent work has addressed the challenges that arise when rare variants are analyzed [3-5].

Most rare variant methods test for a relationship between the disease state or a quantitative trait and the number of mutations in a gene. The statistical test is usually performed by collapsing genotypes across variants that have low frequency, with or without weighting, followed by a univariate test on the aggregate variable. A challenge to overcome in the analysis of rare and common variants jointly is that methods for common variants are suboptimal for the analysis of rare variants, and, conversely, methods proposed for the analysis of rare variants focus essentially on accumulation of rare variants within a given functional unit and are not designed to capture the effect of common variants (i.e., with minor allele frequency [MAF] > 5%). Nevertheless a few methods have been proposed to identify regions that hold both common and rare variants. A haplotype-based approach is one solution when only common variants are available [6], and some general frameworks have been proposed to jointly analyze rare and common variants, as in the combined multivariate and collapsing method [3]. In this last approach, variants are divided and collapsed into subgroups on the basis of allele frequencies, and all subgroups are analyzed jointly using a multivariate test.

In this study, we compare several strategies for analyzing sequence data in the context of an exome-wide association study in which a large number of genes, each of which contains either rare and/or common causal and noncausal variants, have been sequenced in unrelated individuals. We split all genes into subgroups according to the MAF of the SNPs and analyze each subgroup independently using different methods. First, we compare the power and type I error rate of three recently proposed collapsing methods [4,5] when searching for association between a gene and a quantitative phenotype. Second, we explore the advantages and disadvantages of independently analyzing common SNPs (e.g., SNPS that have MAF ≥ 5%) using two different statistical approaches. Third, we combine the results of the rare and common variant tests using Fisher’s method. We compare the power and type I error rate of our combined test with each of the rare and common variant approaches alone. We show, first, that the power of the rare variant approaches to detect the genes harboring multiple causal variants (referred to as causal genes throughout this work) is low in these simulated data and that higher power can be achieve by analyzing common variants only; second, we show that combining signals from rare and common variants can slightly improve the power.

## Methods

### Genetic Analysis Workshop 17 data

In this study we considered the first 100 replicates of quantitative phenotype Q1 in 697 unrelated individuals in the Genetic Analysis Workshop 17 (GAW17) data set [7]. Each individual was genotyped for 24,487 SNPs across 3,205 genes. We separated these 3,205 genes into three groups: (1) genes that have only rare variants (all SNPs with a MAF < 5%), (2) genes that have both rare and common variants (at least one SNP with a MAF ≥ 5% and one SNP with a MAF < 5%), and (3) genes that have only common variants (SNPs with a MAF ≥ 5% only). The quantitative phenotype Q1 was influenced by 39 SNPs in 9 genes. There were 1 to 11 functional variants per gene, with a MAF of 0.07% to 16.5%. Four of these causal genes belong to group 1 (*FLT4*, *HIF1A*, *VEGFA*, and *VEGFC*) and hence had only rare causal variants. The other five genes (*ARNT*, *ELAVL4*, *FLT1*, *HIF1A*, and *KDR*) were all part of group 2. With the exception of *H1F3A*, all genes had at least one causal SNP with a MAF > 1%. Two of them, *KDR* and *FLT1*, also had one causal variant with a MAF > 5%.

### Strategy

We analyzed only the genes of groups 1 and 2 which contain rare variants. Group 1 was analyzed using rare variant methods only. Group 2 was analyzed using the same rare variant methods, then with common variant approaches, and finally with the new combined tests that we defined.

### Analysis of rare variants

We use three rare variant methods that collapse variants from the same gene based on the MAF to build a score. These tests have been recently extended to the analysis of quantitative phenotypes by Price et al. [5]. The three test are (1) the fixed threshold (FT) approach [3], in which all markers that have a MAF below the threshold *T* are collapsed without weighting the markers; (2) the weighted (WE) approach proposed by Madsen and Browning [4], in which all available SNPs are used and weighted inversely to their MAF in control subjects; and (3) the variable threshold (VT) approach [5], which is similar to the previous approaches but searches for the optimal threshold *T*_opt_ that maximizes the score and optionally allows for weighting. The significance level of these three scores is estimated empirically using permutation of the phenotype value. These tests were conducted using the VT Test software (http://genetics.bwh.harvard.edu/vt/dokuwiki/start). The principles of these tests are described in more detail in the GAW17 background papers on collapsing methods [8].

### Analysis of common variants

We compared two approaches to summarize single-SNP associations for each gene *G*. First, we conducted a classic genome-wide analysis of Q1 for all available SNPs that have a MAF over a given threshold *T* using a linear regression under an additive model; we used the minimum *p*-value *P*_min._*_G_* of each gene as the summary statistic. Second, we allowed for simultaneous effects of multiple variants by using the LASSO (least absolute shrinkage and selection operator) algorithm [9] to identify a family of “most predictive” variants. The LASSO, which uses a penalized least-squares criterion, is a computationally fast approach for exploring the options between the additive model that allows separate effects for all variants in the gene and the model that ignores all variants. A sequence of penalty values is defined, and as the penalty decreases, more variants are allowed to enter the model for mean response. A popular tool for choosing the LASSO-based model for a given data set uses an analog [10] of Mallows’s *C_p_*. The LASSO method is described in more detail in the GAW17 background papers on machine learning methods [11]. In our application, we applied the LASSO method separately for each gene to identify a predictive subset of all SNPs in the gene having MAF >*T*. The *p*-value *P*_lasso._*_G_* of the “best (minimum *C_p_*) model” identified by the LASSO method was used as the summary statistic for this approach.

After correcting *P*_min._*_G_* and *P*_lasso._*_G_* for the number of markers in the gene, we can consider these two *p*-values as an association test for the gene. We refer to these two approaches later as CV_pmin_ and CV_lasso_. Note that our correction of *P*_lasso._*_G_* involves multiplication by the total number of SNPs in gene *G*. This is likely conservative because the number of degrees of freedom of a LASSO fit is equal to the number of nonzero coefficients of the fit [10], which is typically much less than the number of SNPs. Further work on calibration of genome-wide inferences with adaptive testing for SNPs is warranted.

### Combined test

Although the VT and WE approaches focus on the aggregation of rare variants, they use all available SNPs from gene *G* to estimate the association with the phenotype and hence are already using information from common SNPs. Thus for the combined test we aggregate the association signal from rare variants derived with the FT approach and the association signal from common variants derived using either CV_pmin_ or CV_lasso_ in the set of SNPs excluded by the FT approach. We used Fisher’s method to combine *p*-values from rare and common variant tests. Fisher’s method is a classical tool for meta-analysis of multiple tests. The principle is to form a single test *U* from a set of *np*-values *p_i_* obtained from *n* independent. *U* is defined as:(1)

Under the null hypothesis of no association of any of the *n* tests, *U* has a chi-square distribution with 2*n* degrees of freedom.

### Comparison of approaches

We first compared type I error rate of the three rare variant approaches and the power to detect the four causal genes of group 1 and the five causal genes of group 2. We used two different MAF thresholds (1% and 5%) for the FT test. We also compared in group 2 the power and type I error rate of the common variant association tests CV_pmin_ and CV_lasso_ for the two thresholds. We then applied the combined test to group 2. Because we defined two statistics to summarize results from the common variant approaches and used two thresholds for the FT test, we applied four combined approaches. For all the tests we conducted, the final *p*-values were corrected for multiple testing using a Bonferroni correction.

### Correction for inflated type I error rate

The first analyses we conducted were subject to a substantial inflation of type I error. We used genomic control [12] to correct all *p*-values. The technique of genomic control consists in measuring the inflation factor *λ* of the distribution of a test statistic, which reflects the difference between the observed distribution and an expected distribution, and dividing all observed values of the tests by this factor. In large-scale analysis, in which most tests are expected to be distributed under the null hypothesis of no association, the inflation factor is usually derived as the ratio between the observed median of the observed test statistic and the expected median under the null hypothesis. The mean of the statistic or of its null distribution can be used instead of the median [13]. In this data set we used the chi-square statistic with 1 degree of freedom corresponding to the observed *p*-value as the observed statistic. The inflation factor *λ* was derived as the ratio of the mean of the observed chi-square statistic over the mean of the expected chi-square statistic under the null hypothesis of no association.

## Results

### Description of the group of genes

The first group of genes included 1,732 genes having only SNPs with MAF < 5%. The mean number of SNPs per gene was 3.155 (SD = 4.116), and the mean frequency of SNPs in this group was 0.006 (SD = 0.009). The second group included 1,142 genes having both rare and common variants (at least one SNP with MAF < 5% and at least one SNP with MAF ≥ 5%). The mean number of SNPs per gene was 16.336 (SD = 20.173), and their frequency on average was 0.035 (SD = 0.088). Group 3 included 331 genes having only variants with MAF ≥ 5%. There was a mean of 1.106 (SD = 0.387) SNPs per gene, and the frequency was 0.186 (SD = 0.124) on average.

### Analysis of all genes without genomic control

**Figure 1:**
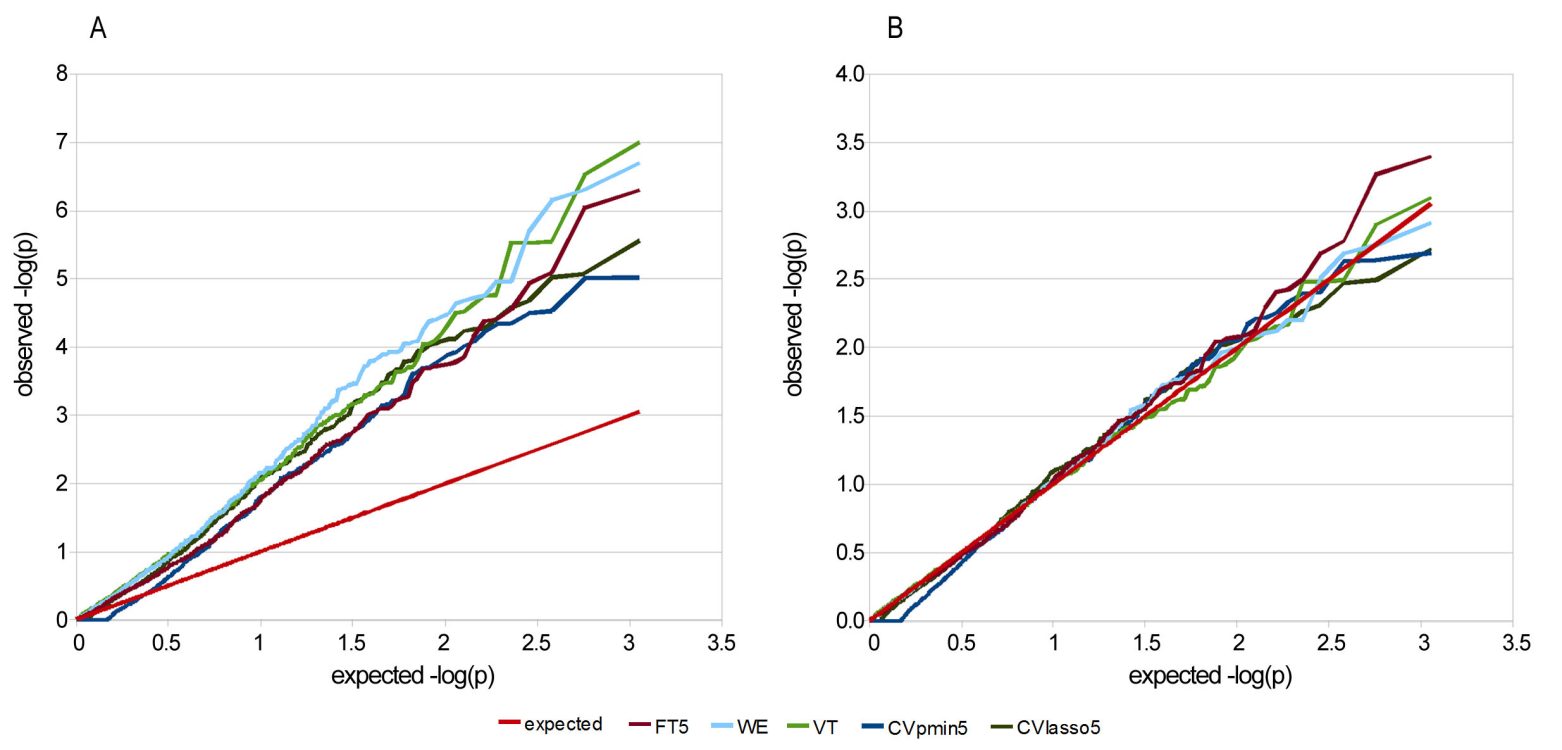
**Quantile-quantile plot of single tests for replicate 1 (A) with and (B) without genomic control adjustment**. FT5, fixed threshold test with MAF threshold equal to 5%; WE, weighted test; VT, variable threshold test; CVpmin5, test of common variants with MAF ≥ 5% using *p*-min; CVlasso5, test of common variants with MAF ≥ 5% using LASSO.

For the overall set of noncausal genes (i.e., all genes that contain no causal SNPs), we first measured the type I error rate of each of the rare variant tests and of the two common variant tests using a threshold *T* of 5%. All these tests were highly inflated with a mean inflation factor *λ* across the 100 replicates of 2.26 (SD = 0.29), 2.75 (SD = 0.36), 2.73 (SD = 0.36), 2.23 (SD = 0.26), and 2.29 (SD = 0.48) for the FT_5%_, WE, VT, CV_pmin,5%_, and CV_lasso,5%_ tests, respectively (those tests that depend on a threshold are indicated with the subscript *T* percentage) (Figure 1A). We used genomic control to correct the *p*-values of all these tests. After correction, all tests showed a correct type I error rate (Figure 1B). Using this adjusted association test, we compared these methods on genes of group 1 and group 2.

### Analysis of genes from group 1

Table 1 shows the power and type I error rate of the VT test, the WE test, and the FT_1%_ and FT_5%_ tests for the genes from group 1. After Bonferroni correction to correct for multiple testing, none of these tests were able to capture any of the four causal genes from group 1. The top ranks and *p*-values of the causal genes among the 1,732 tested genes of this group for replicate 1 were *VEGFC* for the FT_1%_ test (*p* = 0.001, rank = 6), FT_5%_ test (*p* = 0.001, rank = 25), and VT test (*p* = 0.001, rank = 21) and *VEGFA* for the WE test (*p* = 0.0004, rank = 15). Thus an inspection of the top genes will capture only a small fraction of causal genes yet will result in many false positives.

**Table 1:** Comparison of power and type I error rate of single tests and combined tests

Method	Group 1		Group 2	
	
	Power to detect the four causal genes	Type I error rate for *α* = 5%	Power to detect the five causal genes	Type I error rate for *α* = 5%
Rare variant tests				
FT_1%_	0	0.053	0	0.051
FT_5%_	0	0.048	0	0.054
WE	0	0.047	0	0.051
VT	0	0.046	0	0.046
Common variant tests				
CV_pmin,1%_	–	–	0.200	0.052
CV_pmin,5%_	–	–	0.200	0.049
CV_lasso,1%_	–	–	0.094	0.056
CV_lasso,5%_	–	–	0.158	0.059
Combined tests				
Comb_(pmin+FT),1%_	–	–	0.230	0.057
Comb_(pmin+FT),5%_	–	–	0.202	0.058
Comb_(lasso+FT),1%_	–	–	0.182	0.060
Comb_(lasso+FT),5%_	–	–	0.196	0.054

Type I error rate and power were computed over 100 replicates after genomic control adjustment. Group 1 includes genes that have only rare variants; these genes have been analyzed using rare variant approaches only. Group 2 includes genes that have both rare and common variants. FT, fixed threshold test; WE, weighted test; VT, variable threshold test; CV_pmin_, common variants test using *p*-min; CV_lasso_, common variants test using LASSO; Comb, combined test. Tests that depend on a threshold are indicated with the subscript *T* (1% or 5%).

### Analysis of genes from group 2

The second group of genes was analyzed first using the rare variant methods only, then using the common variant tests only, and finally using the combined test (Comb_(pmin+FT)_ for the combined test of CV_pmin_ and FT and Comb_(lasso+FT)_ for the combined test of CV_lasso_ and FT). We considered the two thresholds of 1% and 5%. Power and type I error rate of all these tests are presented in Table 1. As in the analysis of group 1, none of the rare variant tests alone was able to capture a single causal gene over the 100 replicates analyzed. Surprisingly we observed a higher power for the two common variant tests. CV_pmin_ had a power equal to 0.20 whatever threshold was used, and CV_lasso_ had a power of 0.094 and 0.158 for *T* = 1% and *T* = 5%, respectively. Using the combined tests always slightly increased the power to detect the five causal genes of this group compared to the power of the tests considered alone. The highest power (0.23) was observed for the combined approach that used CV_pmin,1%_ and FT_1%_. It is perhaps surprising that CV_pmin_ exhibited greater power than CV_lasso_, but note that the LASSO-based procedure was not forced to include in its minimum *C_p_* the single SNP with minimum *p*-value according to the genetic model used. Alternative approaches to stepwise SNP selection in the common variants context are worthy of investigation.

## Discussion

Genome-wide analysis of sequence data will be a major challenge in complex trait associations over the next several years [14]. Before such studies can be systematized, many technical issues will need to be addressed. In this study we show that the power of recently proposed rare variant methods to detect causal genes may be very low when considered alone but that the association signal from these rare variants can be combined with the association signal of common variants, estimated with classical approaches, resulting in a slight increase in detection ability in our data. Moreover, we show that it will be challenging to identify small genes that have a low number of causal variants in exome-wide association studies. In these simulated data, the power to detect the four genes that had less than four rare causal mutations was null, and the power to detect the five larger genes that contained more mutations was driven by the detection of the genes *KDR* and *FLT1*. These two genes contain a range of causal mutations (from 0.07% to 16%), supporting our strategy, which is based on the combined analysis of both rare and common variants.

In this study we used Fisher’s method to combine *p*-values from rare and common variant analyses. This approach does not take into account potential linkage disequilibrium between rare and common variants; however, the type I error rate of the combined test was not inflated in these data. Other strategies based on permutation analysis can be defined to compute *p*-values of the combined test when linkage disequilibrium is expected.

We compared two thresholds of 1% and 5% for the FT test, the common variant test, and the combined tests. We observed a higher power to detect causal genes when a threshold of 1% was used for the combined test; this seems to indicate that classical approaches, such as linear regression, should be preferred to collapsing approaches when the number of heterozygotes and homozygotes for the rare allele is large enough. Hence the choice of the threshold may depend on the sample size. In this study we analyzed 697 samples, and the minor allele of a SNP with a MAF of at most 1% would be found in a maximum of 14 subjects. Analyzing SNPs with a lower MAF in these data was unreasonable. However, SNPs with a MAF < 1% can be analyzed using regression if the sample size is large enough.

We did not use additional functional information of nonsynonymous SNPs, as proposed by Price et al. [5] using, for example, PolyPhen [15]. Clearly, because the data were simulated using PolyPhen, we would expect an increase in power when incorporating this information. Although this method has been described for the VT test [5], we believe that it can also be added to the combined test to improve the power.

We used genomic control to correct for inflation of type I error rate. Other types of correction or adjustment for population stratification that we conducted (results not shown) were unsuccessful in correcting the type I error. Whether a similar effect will be seen in real data is unclear. Although this correction leads to low power of the rare variant tests, the two causal genes *FLT1* and *KDR* were always in the top 10 of all rare variant tests, including the WE and VT tests. Hence, if the focus of the study is not association testing but rather a screening approach to pick up a set of interesting genes, then these rare variant tests would give results similar to the combined test in these data.

Our analysis of the GAW17 data set is far from exhaustive. Multiple correlated phenotypes generated to represent phenomena of epistasis and gene-environment interaction are present in this simulated data set. Extension of the combined common and rare variant testing procedure to accommodate information on correlated phenotypes and environmental exposures would be of interest.

## Conclusion

The analysis of these simulated data shows that classical approaches, such as linear regression testing common variants, were able to capture more of the association signal between causal genes and the trait of interest than the rare variant tests alone, which was null in all situations we considered. However, combining the association signal from rare and common variants within each gene using Fisher’s method can slightly increase the power to detect the causal genes. The highest power was observed when we combined the minimum *p*-value from the common SNPs and the fixed threshold test of rare SNPs while using a MAF of 1% to separate the rare from the common variants. Nevertheless, considering the low power of the rare variant methods in these data and the strong correction that was applied to all tests to correct the inflation of the type I error rate, the small increase in power that we observed in these data needs to be considered with caution, and more research in this domain is clearly warranted.

## Competing interests

The authors declare that they have no competing interests.

## Authors’ contributions

HA conceived of the study, performed statistical analysis and drafted the manuscript. WQ participated in the design of the study and performed statistical analysis. BP participated in the design of the study and helped draft the manuscript. NZ participated in the design of the study. MHC participated in the design of the study and helped to draft manuscript. VC participated in the design of the study, performed statistical analysis and helped to draft the manuscript. All authors read and approved the final manuscript.
